# Carotid–Femoral Pulse Wave Velocity Could Be a Marker to Predict Cardiovascular and All-Cause Mortality of Hemodialysis Patients

**DOI:** 10.3390/jcm12072509

**Published:** 2023-03-26

**Authors:** Xin-Ning Ng, Jen-Pi Tsai, Chih-Hsien Wang, Bang-Gee Hsu

**Affiliations:** 1 Department of Internal Medicine, Dalin Tzu Chi Hospital, Buddhist Tzu Chi Medical Foundation, Chiayi 62247, Taiwan; 2 Division of Nephrology, Department of Internal Medicine, Dalin Tzu Chi Hospital, Buddhist Tzu Chi Medical Foundation, Chiayi 62247, Taiwan; 3 School of Medicine, Tzu Chi University, Hualien 97004, Taiwan; 4 Division of Nephrology, Hualien Tzu Chi Hospital, Buddhist Tzu Chi Medical Foundation, Hualien 97004, Taiwan

**Keywords:** carotid–femoral pulse wave velocity, arterial stiffness, hemodialysis, mortality

## Abstract

Among hemodialysis (HD) patients, cardiovascular disease (CVD) is recognized as a major contributor to the high risk of mortality, and emerging evidence has ascertained arterial stiffness as an independent predictor of adverse cardiovascular (CV) outcomes. We aimed to investigate the efficacy of arterial stiffness measurement in predicting CV and all-cause mortality in patients on HD (*n* = 130). Carotid–femoral pulse wave velocity (cfPWV) was measured by a validated tonometry system. A cfPWV of >10 m/s was used to assign patients to the arterial stiffness group (*n* = 64). Baseline and biochemical characteristics, as well as all-cause and CV mortality, were recorded. During the 3-year follow-up period, a total of 32 deaths (25%) occurred. The patients who died had clinically significant high cfPWV levels; were relatively old; and had hypoalbuminemia, low creatinine levels, and diabetes. After adjustment for the prognostic variables, patients with elevated cfPWV had significantly higher all-cause (*p* = 0.036) and CV mortality (*p* = 0.017), compared with the mortality rates in the normal group. In this study, cfPWV was found to be an independent predictor of all-cause and CV mortality in HD patients.

## 1. Introduction

Over the past five decades, studies have shown that, compared with the general population, dialysis patients have higher risk of mortality [1,2]. In fact, of all the causes of death, cardiovascular (CV) mortality accounted for 50% among dialysis patients and 26% among those with normal renal function [3,4,5,6]. Therefore, CV disease (CVD) is now recognized as a major contributor to death in dialysis patients, who reportedly had a 10- to 30-fold increased risk of CV mortality, compared with that in the general population [7,8].

Patients with chronic kidney disease (CKD) are predisposed to develop increased arterial stiffness secondary to fibroelastic intimal thickening, elastic lamellae calcification, and increased extracellular matrix deposition, all of which alter arterial wall intrinsic elasticity [9]. Increased arterial stiffness leads to elevated systolic blood pressure, which results in increased pulse pressure, cardiac afterload, and decreased diastolic coronary flow [10,11]. In a cross-sectional study of both central and peripheral arterial stiffness in patients with treatment-naïve essential hypertension, glomerular filtration rate (GFR) and arterial stiffness were found to be inversely related. The augmented arterial stiffness and pulse pressure are, in turn, potentially damaging to the glomerular capillaries and may lead to glomerulosclerosis.

Emerging evidence has ascertained arterial stiffness as an independent predictor of adverse CV outcomes in patients with CKD [12,13,14]. In a study by Sarafadis et al., on patients with advanced CKD, arterial stiffness served as a better prognostic indicator, compared with office and ambulatory measurements of systolic blood pressure [15].

Carotid–femoral pulse wave velocity (cfPWV) has been the gold standard measure of arterial stiffness, given its simplicity in determination, reliability, and substantial evidence supporting its predictive value for CV events [16,17]. In addition, a meta-analysis that enrolled a total of 17,635 subjects showed that cfPWV improved the identification of subjects with high CV risk [18]. Furthermore, the 2020 International Society of Hypertension guidelines for the management of hypertension included cfPWV in the assessment of asymptomatic organ damage [19]. In the present study, we aimed to investigate the efficacy of cfPWV in predicting CV and all-cause mortality in patients on hemodialysis (HD).

## 2. Materials and Methods

### 2.1. Participants 

This single-center observational longitudinal study was conducted from January to April 2014 and enrolled patients who were >20 years old and were on chronic HD for more than 3 months, with the following specifications: standard 4-hour HD, three times a week, and use of high-flux polysulfone disposable artificial kidneys (FX class dialyzer; Fresenius Medical Care, Bad Homburg, Germany). A total of 130 patients were enrolled in this study. All participants provided informed consent prior to study participation. This study was approved by the research ethics committees of Hualien Tzu Chi Hospital, Buddhist Tzu Chi Medical Foundation (IRB103-136-B). Patients who had acute infection, malignancy, amputations, acute heart failure, and a life expectancy of less than 6 months at the time of blood sampling, as well as those who refused to provide informed consent, were excluded from this study. Systolic blood pressure (SBP) and diastolic blood pressure (DBP) were measured three times at 5-minute intervals; the average values were used for further analysis. SBP ≥ 140 mmHg and/ or DBP ≥ 90 mmHg or receipt of any antihypertensive medications in the past 2 weeks was defined as hypertension. Diabetes mellitus (DM) was defined as a fasting plasma glucose of ≥126 mg/dL or intake of oral hypoglycemic medications or insulin. To further analyze the relationships of all the clinical and biochemical variables with arterial stiffness and mortality, the patients were classified into the arterial stiffness and control group, based on the cf-PWV cut-off threshold of 10 m/s.

### 2.2. Anthropometric Analysis

Body weight and height were measured to the closest half kilogram and half centimeter, respectively, wearing light clothing but without shoes. The post-HD body weight was used to determine the body mass index (kg/m^2^) [20,21].

### 2.3. Biochemical Investigations

Before HD, 5 mL of blood were collected. The remaining blood sample was centrifuged for biochemical analyses after the hemoglobin level was determined (Sysmex SP-1000i; Sysmex American, Mundelein, IL, USA). These analyses included total cholesterol, triglycerides (TG), glucose, blood urea nitrogen, creatinine, calcium, phosphorus, and albumin (Sysmex SP-1000i; SiemensAdvia 1800, Siemens Healthcare GmbH, Henkestr, Germany). Intact parathyroid hormone level (iPTH) was analyzed using a commercially available enzyme-linked immunosorbent assay (IBL International GmbH, Hamburg, Germany) [20,21]. The fractional clearance index for urea (Kt/V) and urea reduction ratio were calculated in a single compartment dialysis urea kinetic model.

### 2.4. Carotid–Femoral Pulse Wave Velocity Measurements

Measurements of cfPWV were obtained after at least 10 min of rest, using pressure applanation tonometry (SphygmoCor system; AtCor Medical, New South Wales, Australia), as detailed in previous studies [20,21]. Arterial stiffness was defined as cfPWV >10 m/s, according to the European Society of Hypertension and the European Society of Cardiology 2018 guidelines [22].

### 2.5. Follow-Up and Endpoints

Patient conditions were monitored for 36 months until 30 June 2017 during follow-up visits at the HD room and based on medical records. The study endpoint was all-cause mortality. The causes of mortality were hemorrhagic stroke (*n =* 7), ischemic stroke (*n =* 10), acute myocardial infarction (*n =* 5), and septic shock (*n =* 5). CV mortality comprised hemorrhagic stroke, ischemic stroke, or acute myocardial infarction. Event-free survival was defined as the interval between cfPWV evaluation and death or the end of study follow-up.

### 2.6. Statistical Analysis

Continuous variables were evaluated for normal distribution using the Kolmogorov–Smirnov test. The two-tailed independent Student’s t-test was used to compare the mean and standard deviation of the variables with normal distribution between the groups. Using the Mann–Whitney U test, nonnormally distributed variables (such as HD vintage, TG, glucose, albumin, and iPTH) were reported as median and interquartile range and compared between the groups. Categorical variables were quantified as a number (%), and the chi-square test was used for analysis. Kaplan–Meier curves on all-cause and CV mortality were used to illustrate the survival of the HD patients on follow-up analyses. The cumulative proportion of HD patients who were free from all-cause or CV death was compared between groups using a log-rank test. The risk factors for all-cause and CV mortality were investigated using multivariate Cox regression models. SPSS for Windows was used to examine the data (version 19.0; SPSS Inc., Chicago, IL, USA). A *p* value of <0.05 was considered statistically significant.

## 3. Results

Among the enrolled patients (*n* = 130), 64 were classified to the arterial stiffness group and 66 were classified to the control group, based on the cf-PWV cut-off threshold of 10 m/s. The baseline characteristics of the cohort are presented in Table 1. The mean age was 63 years and 47.7% were women. The average HD vintage was 55.9 months; 40% had DM and 53% had hypertension. Compared with the control group, the arterial stiffness group was significantly older (*p* = 0.047) and had significantly higher SBP (*p* = 0.003), serum glucose level (*p* = 0.020), prevalence of DM (*p* < 0.001) and hypertension (*p* = 0.034), and proportion of patients receiving statin treatment (*p* = 0.043).

**Figure 1:**
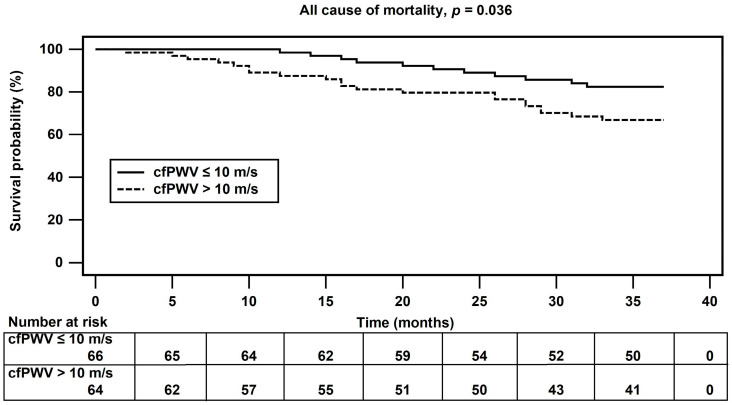
Kaplan–Meier analysis of carotid–femoral pulse wave velocity levels and all-cause mortality events in hemodialysis patients.

During the 3-year follow-up period, a total of 32 deaths (25%) occurred, and 22 (16.9%) were CV deaths. Compared with patients who survived, those who died were significantly older (*p* = 0.007) and had significantly higher cfPWV levels (*p* < 0.001), lower serum albumin (*p* = 0.003) and creatinine (*p* < 0.001) levels, and higher prevalence of DM (*p* = 0.003) (Table 2). After adjustment for the prognostic variables (Cox Model 3), the hazard ratio (HR) for all-cause mortality per 1 m/s increase in cfPWV was 1.17 (95% CI 1.033–1.326, *p* = 0.013) (Table 3). Compared with the control group, the arterial stiffness group had significantly higher all-cause mortality (*p* = 0.036), as shown in Figure 1.

**Figure 2:**
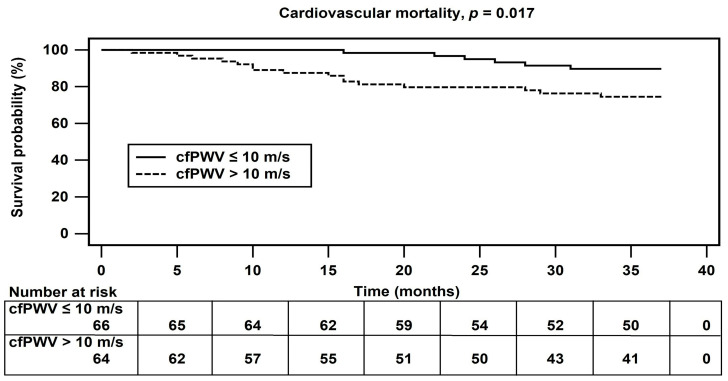
Kaplan–Meier analysis of carotid–femoral pulse wave velocity levels and cardiovascular mortality events in hemodialysis patients.

Compared with patients who survived, those who died from CV causes had significantly higher levels of cfPWV (*p* < 0.001), lower serum creatinine (*p* = 0.020), and higher prevalence of DM (*p* = 0.001) (Table 4). On multivariate Cox regression analysis with adjustment for prognostic variables, increased cfPWV level was significantly associated with CV death (Cox Model 3, HR 1.317, 95% CI 1.13–1.534; *p* < 0.001) (Table 5). The Kaplan–Meier plot in Figure 2 illustrates that an elevated cfPWV of >10 m/s was associated with increased CV mortality in HD patients (*p* = 0.017).

## 4. Discussion

After controlling for other significant factors in this longitudinal observational analysis, we found that cfPWV had a substantial predictive value for all-cause and CV mortality in patients on HD.

CKD is associated with a chronic inflammatory state and oxidative stress, which promote arterial intima media calcification and results in increased arterial stiffness [23,24]. Aortic compliance decreases with the progression of CKD, thereby leading to higher SBP and pulse pressure [23,24]. The augmented pulsatility results in accelerated pulse wave transmission through the large arterial walls, as represented by the level of cfPWV [25]. A high SBP increases cardiac afterload, left ventricular contractile effort, and myocardial oxygen consumption [26].

The cfPWV is currently regarded as the gold standard measure of arterial stiffness, because it was demonstrated to correlate with mortality and was more predictive of CV mortality compared with femorotibial PWV or carotid–radial PWV in a cohort study on patients with end-stage renal disease (ESRD) [17,27,28]. Moreover, mounting evidence has demonstrated cfPWV as a surrogate marker of future CV events [17,27,28]. In a meta-analysis, participants who had high cfPWV by 1 m/s had high pooled relative risks for CVD events (HR 1.12, 95% CI 1.07–1.18) and CVD mortality (HR 1.09, 95% CI 1.04–1.14) [29]. At the cut-off point of 10 m/s, cfPWV was identified as a prognostic indicator of death and rehospitalization (hazard ratio of 1.7) in patients hospitalized with acute decompensated heart failure.

Another meta-analysis that included 15,877 participants showed that the relative risk of CV and all-cause mortality increased by 15% when the cfPWV increased by 1 m/s [30]. Moreover, cfPWV was shown to predict CV events better than conventional risk factors [18]. Another systematic review and meta-analysis indicated that cfPWV as a predictor had good accuracy for CV mortality but slightly lower accuracy for all-cause mortality, with area under the hierarchical summary receiver operating characteristic curve values of 0.75 (95% CI 0.69–0.81) and 0.78 (95% CI 0.74–0.83), respectively, and the closest cutoff point to the summary point values of 10.7 and 11.5, respectively [31].

In the Chronic Renal Insufficiency Cohort study, which enrolled 2795 participants with CKD (i.e., mean eGFR of 44 mL/min/1.73 m^2^) and included 47.3% with DM, cfPWV was identified as a significant predictor of mortality and CKD progression within a mean follow-up period of 5 years; moreover, those in the highest tertile of cfPWV (i.e., >10.3 m/s) had increased mortality risk (HR 1.72, 95% CI 1.24–2.38) [32]. In a multicenter longitudinal study on 2602 patients with CKD, cfPWV was found to be a significant predictor of incident heart failure hospitalization, with those in the highest tertile of cfPWV having an HR of 3.01 (95% CI 1.45–6.26), compared with that in cases in the lowest tertile [33]. In another longitudinal study on 305 patients with ESRD, cfPWV was shown to have a prognostic value for CV mortality; using a cutoff value of 10.75 m/s, the area under the receiver operating characteristic curve value was 83.4 ± 2.3, with 84% sensitivity, 73% specificity, 87.3% negative predictive value, and 72% positive predictive value [14]. In a cohort of 1084 HD patients recruited from 47 European dialysis centers, each 1 m/s increase in cfPWV was found to be associated with a 15% higher risk of CV events (HR 1.154, 95% CI 1.085–1.228) after a median follow-up of 2 years [34]. In a French cohort study on HD patients (*n* = 287), each 1 m/s increase in cfPWV was associated with 17% higher risk of CV mortality (HR 1.17, 95% CI 1.07–1.28) and 10% higher risk of all-cause mortality (HR 1.10, 95% CI 1.02–1.20) [35]. However, a cohort study in Canada on HD patients (*n* = 246) did not find an association between each 1 m/s increase in cfPWV and CV mortality or all-cause mortality after adjustment for confounders [12]. Another study suggested that cfPWV had a prognostic value for all-cause and CV mortality, although inferior to simple clinical risk scores (i.e., annualized rate of occurrence cohort), only modestly improved the risk discrimination and reclassification by the same risk scores, and worsened the model calibrations [36]. In a longitudinal cohort study on 150 patients with ESRD, reduction in PWV levels with the use of antihypertensive drugs had a beneficial effect on survival [37]. However, the beneficial effect of antihypertensives remains speculative. In this study, we similarly found that elevated cfPWV was associated with CV and all-cause mortality, after adjusting for prognostic variables, in patients on HD.

Our study had several limitations. First, the single-center observational design and the setting in Taiwan only may limit the generalizability to all patients with CKD, those on peritoneal dialysis, or those in different areas. Second, the number of HD patients was limited and the follow-up period was only 36 months. In addition, many other factors affect the risk of CV or all-cause mortality in patients undergoing HD [38]. Therefore, further longitudinal studies with a large number of HD patients are needed to clarify our findings.

## 5. Conclusions

In this study, cfPWV was found to be an independent predictor of all-cause and CV mortality in patients on HD. Therefore, we propose cfPWV as a potential modifiable risk factor in this population. Finally, future validation of various interventions is required to improve cfPWV measurements and decrease CV events.

## Figures and Tables

**Table 1:** Clinical variables of arterial stiffness group or control group.

Variables	All Participants (*n* = 130)	Control Group (*n* = 66)	Arterial Stiffness Group (*n* = 64)	*p* Value
Age (years)	63.17 ± 12.94	60.95 ± 12.97	65.45 ± 12.59	0.047 *
HD vintage (months)	55.92 (23.40–115.68)	60.06 (21.99–136.80)	54.54 (24.99–99.30)	0.325
Height (cm)	160.47 ± 8.19	160.23 ± 8.46	160.72 ± 7.97	0.734
Pre-HD body weight (kg)	64.49 ± 14.63	63.76 ± 15.38	65.24 ± 13.90	0.566
Post-HD body weight (kg)	62.32 ± 14.12	61.68 ± 14.74	62.98 ± 13.54	0.601
Body mass index (kg/m^2^)	25.11 ± 4.81	24.88 ± 5.07	25.35 ± 4.56	0.573
Carotid-femoral PWV (m/s)	10.43 ± 3.28	7.82 ± 1.23	13.13 ± 2.44	<0.001 *
Systolic blood pressure (mmHg)	143.27 ± 26.58	136.58 ± 26.31	150.17 ± 25.24	0.003 *
Diastolic blood pressure (mmHg)	77.47 ± 16.35	76.68 ± 15.48	78.28 ± 17.29	0.579
Hemoglobin (g/dL)	10.45 ± 1.12	10.27 ± 1.21	10.63 ± 1.00	0.067
Total cholesterol (mg/dL)	145.83 ± 34.61	149.61 ± 38.78	141.94 ± 29.51	0.208
Triglyceride (mg/dL)	118.00 (87.00–185.50)	113.00 (84.00–205.50)	124.50 (89.25–175.75)	0.872
Albumin (g/dL)	4.10 (3.90–4.40)	4.20 (3.98–4.40)	4.10 (3.90–4.30)	0.214
Glucose (mg/dL)	131.00 (108.50–169.00)	126.50 (103.00–148.75)	138.00 (114.25–185.00)	0.020 *
Blood urea nitrogen (mg/dL)	61.36 ± 13.80	61.27 ± 13.53	61.45 ± 14.17	0.941
Creatinine (mg/dL)	9.55 ± 2.04	9.76 ± 2.09	9.33 ± 1.99	0.239
Total calcium (mg/dL)	9.03 ± 0.79	8.94 ± 0.78	9.12 ± 0.80	0.209
Phosphorus (mg/dL)	4.72 ± 1.29	4.67 ± 1.33	4.77 ± 1.26	0.644
iPTH (pg/mL)	197.95 (69.38–440.68)	245.95 (102.40–375.85)	161.70 (57.28–463.85)	0.299
Urea reduction rate	0.73 ± 0.04	0.73 ± 0.04	0.73 ± 0.04	0.987
Kt/V (Gotch)	1.34 ± 0.17	1.34 ± 0.17	1.34 ± 0.16	0.935
Female, *n* (%)	62 (47.7)	33 (50.0)	29 (45.3)	0.593
Diabetes mellitus, *n* (%)	52 (40.0)	13 (19.7)	39 (60.9)	<0.001 *
Hypertension, *n* (%)	69 (53.1)	29 (43.9)	40 (62.5)	0.034 *
ARB, *n* (%)	37 (28.5)	18 (27.3)	19 (29.7)	0.760
β-blocker, *n* (%)	41 (31.5)	20 (30.3)	21 (32.8)	0.758
CCB, *n* (%)	51 (39.2)	29 (43.9)	22 (34.4)	0.264
Statin, *n* (%)	20 (15.4)	6 (9.1)	14 (21.9)	0.043 *
Fibrate, *n* (%)	16 (12.3)	9 (13.6)	7 (10.9)	0.640

HD: hemodialysis; PWV: pulse wave velocity; iPTH: intact parathyroid hormone; Kt/V: fractional clearance index for urea; ARB: angiotensin-receptor blocker; CCB: calcium-channel blocker. * *p* < 0.05 was considered statistically significant.

**Table 2:** Clinical variables of the 130 hemodialysis patients with or without mortality.

Variables	Participants without Mortality (*n* = 98)	Participants with Mortality (*n* = 32)	*p* Value
Age (years)	61.43 ± 12.95	68.50 ± 11.52	0.007 *
HD vintage (months)	57.00 (21.69–125.10)	51.48 (26.43–81.43)	0.693
Height (cm)	161.08 ± 8.50	158.59 ± 6.96	0.136
Pre-HD body weight (kg)	65.59 ± 14.18	61.12 ± 15.69	0.134
Post-HD body weight (kg)	63.34 ± 13.71	59.18 ± 15.09	0.149
Body mass index (kg/m^2^)	25.30 ± 4.70	24.54 ± 5.18	0.440
Carotid-femoral PWV (m/s)	9.67 ± 2.69	12.75 ± 3.85	<0.001 *
Systolic blood pressure (mmHg)	142.64 ± 25.35	145.19 ± 30.39	0.640
Diastolic blood pressure (mmHg)	78.59 ± 16.51	74.03 ± 15.60	0.172
Hemoglobin (g/dL)	10.45 ± 1.15	10.45 ± 1.07	0.982
Total cholesterol (mg/dL)	149.03 ± 34.94	136.03 ± 32.13	0.065
Triglyceride (mg/dL)	119.50 (88.50–197.75)	106.50 (87.00–140.00)	0.218
Albumin (g/dL)	4.20 (4.00–4.50)	4.10 (3.70–4.18)	0.003 *
Glucose (mg/dL)	128.50 (103.75–162.00)	143.00 (120.75–184.00)	0.060
Blood urea nitrogen (mg/dL)	62.12 ± 13.34	59.03 ± 15.08	0.273
Creatinine (mg/dL)	9.93 ± 1.94	8.38 ± 1.94	<0.001 *
Total calcium (mg/dL)	9.08 ± 0.79	8.89 ± 0.80	0.233
Phosphorus (mg/dL)	4.78 ± 1.29	4.53 ± 1.27	0.342
iPTH (pg/mL)	200.25 (73.05–416.65)	189.40 (60.48–466.90)	0.791
Urea reduction rate	0.73 ± 0.04	0.74 ± 0.05	0.380
Kt/V (Gotch)	1.33 ± 0.16	1.36 ± 0.19	0.346
Female, *n* (%)	48 (49.0)	14 (43.8)	0.607
Diabetes mellitus, *n* (%)	32 (32.7)	20 (62.5)	0.003 *
Hypertension, *n* (%)	50 (51.0)	19 (59.4)	0.411
ARB, *n* (%)	28 (28.6)	9 (28.1)	0.961
β-blocker, *n* (%)	33 (33.7)	8 (25.0)	0.359
CCB, *n* (%)	42 (42.9)	9 (28.1)	0.138
Statin, *n* (%)	17 (17.3)	3 (9.4)	0.278
Fibrate, *n* (%)	12 (12.2)	4 (12.5)	0.970

HD: hemodialysis; PWV: pulse wave velocity; iPTH: intact parathyroid hormone; Kt/V: fractional clearance index for urea; ARB: angiotensin-receptor blocker; CCB: calcium-channel blocker. * *p* < 0.05 was considered statistically significant.

**Table 3:** Hazard ratio for all-cause mortality events by multivariable Cox regression of carotid-femoral pulse wave velocity levels among the 130 hemodialysis patients.

Carotid-Femoral PWV (m/s)	Unadjusted		Model 1		Model 2		Model 3	
	HR (95% CI)	*p* Value	HR (95% CI)	*p* Value	HR (95% CI)	*p* Value	HR (95% CI)	*p* Value
Per 1 m/s carotid-femoral PWV increase	1.267 (1.144–1.403)	<0.001 *	1.241 (1.120–1.377)	<0.001 *	1.179 (1.044–1.332)	0.008 *	1.170 (1.033–1.326)	0.013 *

Model 1 is adjusted for age, gender, and body mass index. Model 2 is adjusted using variables, including those in Model 1, along with diabetes mellitus, hypertension, fasting glucose, total cholesterol, and triglyceride. Model 3 is adjusted using variables, including those in Model 2, along with albumin, blood urea nitrogen, creatinine, and hemodialysis vintage. PWV: pulse wave velocity; HR: hazard ratio; CI: confidence interval. * *p* < 0.05 was considered statistically significant after Cox regression analysis.

**Table 4:** Clinical variables of the 130 hemodialysis patients with or without cardiovascular mortality.

Variables	Participants without CV Mortality (*n* = 108)	Participants with CV Mortality (*n* = 22)	*p* Value
Age (years)	62.39 ± 13.37	67.00 ± 9.95	0.128
HD vintage (months)	56.52 (22.23–123.60)	51.48 (27.69–81.21)	0.675
Height (cm)	160.42 ± 8.64	160.73 ± 5.62	0.872
Pre-HD body weight (kg)	64.45 ± 14.34	64.68 ± 16.33	0.946
Post-HD body weight (kg)	62.26 ± 13.84	62.58 ± 15.75	0.924
Body mass index (kg/m^2^)	25.06 ± 4.65	25.37 ± 5.64	0.787
Carotid-femoral PWV (m/s)	9.75 ± 2.78	13.78 ± 3.56	<0.001 *
Systolic blood pressure (mmHg)	142.87 ± 26.12	145.23 ± 29.30	0.706
Diastolic blood pressure (mmHg)	77.96 ± 16.47	75.04 ± 15.90	0.448
Hemoglobin (g/dL)	10.46 ± 1.12	10.41 ± 1.18	0.850
Total cholesterol (mg/dL)	147.36 ± 34.74	138.32 ± 33.75	0.266
Triglyceride (mg/dL)	119.50 (87.75–195.00)	106.50 (71.75–160.75)	0.290
Albumin (g/dL)	4.10 (3.93–4.40)	4.10 (3.70–4.20)	0.079
Glucose (mg/dL)	128.50 (104.25–162.00)	148.50 (124.50–182.00)	0.059
Blood urea nitrogen (mg/dL)	61.56 ± 13.68	60.41 ± 14.66	0.724
Creatinine (mg/dL)	9.74 ± 2.02	8.63 ± 1.96	0.020 *
Total calcium (mg/dL)	9.06 ± 0.79	8.90 ± 0.80	0.406
Phosphorus (mg/dL)	4.71 ± 1.29	4.75 ± 1.33	0.896
iPTH (pg/mL)	195.20 (57.53–401.13)	230.65 (88.10–577.33)	0.203
Urea reduction rate	0.74 ± 0.04	0.73 ± 0.05	0.445
Kt/V (Gotch)	1.34 ± 0.16	1.31 ± 0.18	0.454
Female, *n* (%)	54 (50.0)	8 (36.4)	0.243
Diabetes mellitus, *n* (%)	36 (33.3)	16 (72.7)	0.001 *
Hypertension, *n* (%)	54 (50.0)	15 (68.2)	0.119
ARB, *n* (%)	31 (28.7)	6 (27.3)	0.892
β-blocker, *n* (%)	33 (30.6)	8 (36.4)	0.593
CCB, *n* (%)	44 (40.7)	7 (31.8)	0.435
Statin, *n* (%)	17 (15.7)	3 (13.6)	0.803
Fibrate, *n* (%)	14 (13.0)	2 (9.1)	0.614

CV: cardiovascular; HD: hemodialysis; PWV: pulse wave velocity; iPTH: intact parathyroid hormone; Kt/V: fractional clearance index for urea; ARB: angiotensin-receptor blocker; CCB: calcium-channel blocker. * *p* < 0.05 was considered statistically significant.

**Table 5:** Hazard ratio for cardiovascular mortality events by multivariable Cox regression of carotid–femoral pulse wave velocity levels among the 130 hemodialysis patients.

Carotid-Femoral PWV (m/s)	Unadjusted		Model 1		Model 2		Model 3	
	HR (95% CI)	*p* Value	HR (95% CI)	*p* Value	HR (95% CI)	*p* Value	HR (95% CI)	*p* Value
Per 1 m/s carotid-femoral PWV increase	1.390 (1.234–1.567)	<0.001 *	1.396 (1.227–1.588	<0.001 *	1.336 (1.149–1.552)	<0.001 *	1.317 (1.130–1.534)	<0.001 *

Model 1 is adjusted for age, gender, and body mass index. Model 2 is adjusted using variables including those in Model 1, along with diabetes mellitus, hypertension, fasting glucose, total cholesterol, and triglyceride. Model 3 is adjusted using variables including those in Model 2, along with albumin, blood urea nitrogen, creatinine, and hemodialysis vintage. PWV: pulse wave velocity; HR: hazard ratio; CI: confidence interval. * *p* < 0.05 was considered statistically significant after Cox regression analysis.

## Data Availability

The data presented in this study are available on request from the corresponding author.

## References

[1] Bloembergen W.E., Port F.K., Mauger E.A., Wolfe R.A. (1994). Causes of death in dialysis patients: Racial and gender differences. J. Am. Soc. Nephrol..

[2] Lindner A., Charra B., Sherrard D.J., Scribner B.H. (1974). Accelerated atherosclerosis in prolonged maintenance hemodialysis. N. Engl. J. Med..

[3] Cheung A.K., Sarnak M.J., Yan G., Berkoben M., Heyka R., Kaufman A., Lewis J., Rocco M., Toto R., Windus D. (2004). Cardiac diseases in maintenance hemodialysis patients: Results of the HEMO Study. Kidney Int..

[4] Foley R.N., Parfrey P.S., Sarnak M.J. (1998). Clinical epidemiology of cardiovascular disease in chronic renal disease. Am. J. Kidney Dis..

[5] Foley R.N., Parfrey P.S., Sarnak M.J. (1998). Epidemiology of cardiovascular disease in chronic renal disease. J. Am. Soc. Nephrol..

[6] Jankowski J., Floege J., Fliser D., Böhm M., Marx N. (2021). Cardiovascular disease in chronic kidney disease. Circulation.

[7] Collins A.J., Foley R.N., Gilbertson D.T., Chen S.C. (2015). United States Renal Data System public health surveillance of chronic kidney disease and end-stage renal disease. Kidney Int. Suppl..

[8] Carrero J.J., de Jager D.J., Verduijn M., Ravani P., De Meester J., Heaf J.G., Finne P., Hoitsma A.J., Pascual J., Jarraya F. (2011). Cardiovascular and noncardiovascular mortality among patients starting dialysis. Clin. J. Am. Soc. Nephrol..

[9] Guérin A.P., Pannier B., Métivier F., Marchais S.J., London G.M. (2008). Assessment and significance of arterial stiffness in patients with chronic kidney disease. Curr. Opin. Nephrol. Hypertens..

[10] Angoff R., Mosarla R.C., Tsao C.W. (2021). Aortic stiffness: Epidemiology, risk factors, and relevant biomarkers. Front. Cardiovasc. Med..

[11] Cavalcante J.L., Lima J.A., Redheuil A., Al-Mallah M.H. (2011). Aortic stiffness: Current understanding and future directions. J. Am. Coll. Cardiol..

[12] Fortier C., Mac-Way F., Desmeules S., Marquis K., De Serres S.A., Lebel M., Boutouyrie P., Agharazii M. (2015). Aortic-brachial stiffness mismatch and mortality in dialysis population. Hypertension.

[13] Karras A., Haymann J.P., Bozec E., Metzger M., Jacquot C., Maruani G., Houillier P., Froissart M., Stengel B., Guardiola P. (2012). Large artery stiffening and remodeling are independently associated with all-cause mortality and cardiovascular events in chronic kidney disease. Hypertension.

[14] Pannier B., Guérin A.P., Marchais S.J., Safar M.E., London G.M.J.H. (2005). Stiffness of capacitive and conduit arteries: Prognostic significance for end-stage renal disease patients. Hypertension.

[15] Sarafidis P.A., Loutradis C., Karpetas A., Tzanis G., Piperidou A., Koutroumpas G., Raptis V., Syrgkanis C., Liakopoulos V., Efstratiadis G. (2017). Ambulatory pulse wave velocity is a stronger predictor of cardiovascular events and all-cause mortality than office and ambulatory blood pressure in hemodialysis patients. Hypertension.

[16] Reference Values for Arterial Stiffness ‘Collaboration (2010). Determinants of pulse wave velocity in healthy people and in the presence of cardiovascular risk factors: ’establishing normal and reference values’. Eur. Heart J..

[17] Laurent S., Boutouyrie P., Asmar R., Gautier I., Laloux B., Guize L., Ducimetiere P., Benetos A. (2001). Aortic stiffness is an independent predictor of all-cause and cardiovascular mortality in hypertensive patients. Hypertension.

[18] Ben-Shlomo Y., Spears M., Boustred C., May M., Anderson S.G., Benjamin E.J., Boutouyrie P., Cameron J., Chen C.H., Cruickshank J.K. (2014). Aortic pulse wave velocity improves cardiovascular event prediction: An individual participant meta-analysis of prospective observational data from 17,635 subjects. J. Am. Coll. Cardiol..

[19] Unger T., Borghi C., Charchar F., Khan N.A., Poulter N.R., Prabhakaran D., Ramirez A., Schlaich M., Stergiou G.S., Tomaszewski M. (2020). 2020 International society of hypertension global hypertension practice guidelines. Hypertension.

[20] Hou J.S., Wang C.H., Lai Y.H., Kuo C.H., Lin Y.L., Hsu B.G., Tsai J.P. (2020). Serum malondialdehyde-modified low-density lipoprotein is a risk factor for central arterial stiffness in maintenance hemodialysis patients. Nutrients.

[21] Chiu L.T., Hung C.D., Lin L., Lin Y.L., Hsu B.G. (2022). Serum fibroblast growth factor 21 level is associated with aortic stiffness in patients on maintenance hemodialysis. Int. J. Hypertens..

[22] Williams B., Mancia G., Spiering W., Agabiti Rosei E., Azizi M., Burnier M., Clement D.L., Coca A., de Simone G., Dominiczak A. (2018). 2018 ESC/ESH guidelines for the management of arterial hypertension. Eur. Heart J..

[23] Lioufas N., Hawley C.M., Cameron J.D., Toussaint N.D. (2019). Chronic kidney disease and pulse wave velocity: A narrative review. Int. J. Hypertens..

[24] Tsai J.P., Hsu B.G. (2020). Arterial stiffness: A brief review. Tzu Chi Med. J..

[25] Zhang Y., Lacolley P., Protogerou A.D., Safar M.E. (2020). Arterial stiffness in hypertension and function of large arteries. Am. J. Hypertens..

[26] Inserra F., Forcada P., Castellaro A., Castellaro C. (2021). Chronic kidney disease and arterial stiffness: A two-way path. Front. Med..

[27] Blacher J., Safar M.E., Guerin A.P., Pannier B., Marchais S.J., London G.M. (2003). Aortic pulse wave velocity index and mortality in end-stage renal disease. Kidney Int..

[28] Sutton-Tyrrell K., Najjar S.S., Boudreau R.M., Venkitachalam L., Kupelian V., Simonsick E.M., Havlik R., Lakatta E.G., Spurgeon H., Kritchevsky S. (2005). Elevated aortic pulse wave velocity, a marker of arterial stiffness, predicts cardiovascular events in well-functioning older adults. Circulation.

[29] Zhong Q., Hu M.J., Cui Y.J., Liang L., Zhou M.M., Yang Y.W., Huang F. (2018). Carotid-femoral pulse wave velocity in the prediction of cardiovascular events and mortality: An updated systematic review and meta-analysis. Angiology.

[30] Vlachopoulos C., Aznaouridis K., Stefanadis C. (2010). Prediction of cardiovascular events and all-cause mortality with arterial stiffness: A systematic review and meta-analysis. J. Am. Coll. Cardiol..

[31] Sequí-Domínguez I., Cavero-Redondo I., Álvarez-Bueno C., Pozuelo-Carrascosa D.P., Nuñez de Arenas-Arroyo S., Martínez-Vizcaíno V. (2020). Accuracy of pulse wave velocity predicting cardiovascular and all-cause mortality. A systematic review and meta-analysis. J. Clin. Med..

[32] Townsend R.R., Anderson A.H., Chirinos J.A., Feldman H.I., Grunwald J.E., Nessel L., Roy J., Weir M.R., Wright J.T., Bansal N. (2018). Association of pulse wave velocity with chronic kidney disease progression and mortality: Findings from the CRIC study (Chronic Renal Insufficiency Cohort). Hypertension.

[33] Chirinos J.A., Khan A., Bansal N., Dries D.L., Feldman H.I., Ford V., Anderson A.H., Kallem R., Lash J.P., Ojo A. (2014). Arterial stiffness, central pressures, and incident hospitalized heart failure in the chronic renal insufficiency cohort study. Circ. Heart Fail..

[34] Verbeke F., Van Biesen W., Honkanen E., Wikström B., Jensen P.B., Krzesinski J.M., Rasmussen M., Vanholder R., Rensma P.L., CORD Study Investigators (2011). Prognostic value of aortic stiffness and calcification for cardiovascular events and mortality in dialysis patients: Outcome of the calcification outcome in renal disease (CORD) study. Clin. J. Am. Soc. Nephrol..

[35] Blacher J., Guerin A.P., Pannier B., Marchais S.J., Safar M.E., London G.M. (1999). Impact of aortic stiffness on survival in end-stage renal disease. Circulation.

[36] Tripepi G., Agharazii M., Pannier B., D’Arrigo G., Mallamaci F., Zoccali C., London G. (2018). Pulse wave velocity and prognosis in end-stage kidney disease. Hypertension.

[37] Guerin A.P., Blacher J., Pannier B., Marchais S.J., Safar M.E., London G.M. (2001). Impact of aortic stiffness attenuation on survival of patients in end-stage renal failure. Circulation.

[38] Ma L., Zhao S. (2017). Risk factors for mortality in patients undergoing hemodialysis: A systematic review and meta-analysis. Int. J. Cardiol..

